# Prevalence of genital *Chlamydia trachomatis* infection among young men and women in Spain

**DOI:** 10.1186/1471-2334-13-388

**Published:** 2013-08-22

**Authors:** Carlos Fernández-Benítez, Patricia Mejuto-López, Luis Otero-Guerra, Mario Juan Margolles-Martins, Pilar Suárez-Leiva, Fernando Vazquez

**Affiliations:** 1 Unidad de Gestión Clínica Centro de Salud de Laviana, Asturias, Spain; 2 Hospital Universitario Central de Asturias, Oviedo, Spain; 3 Hospital de Cabueñes, Los Prados 395, Gijón 33396, Spain; 4 Consejería de Sanidad, Asturias, Spain; 5 Departamento Biología Funcional, Área de Microbiología, Facultad de Medicina, Oviedo, Spain

**Keywords:** Sexually transmitted diseases, Chlamydia infections, Prevalence, Mass screening

## Abstract

**Background:**

There are no accurate data regarding the real prevalence of *Chlamydia trachomatis* infection in Spain. Our aim was to determine the prevalence of *C. trachomatis* infections and the risk factors for acquiring them among 1,048 young (15–24 years old) inhabitants of Laviana.

**Methods:**

The study was completed in the period between 1^st^ November 2010 and 31^st^ December 2011. We conducted a capture strategy in the whole population, instead of only in a sample group, with a capture conducted in schools, in the local health centre, by post and by phone as a last resort. The design was based on the model used by Shafer to increase screening rates. *C. trachomatis* was identified by RT-PCR in urine samples.

**Results:**

A total of 487 sexually active people underwent the test, which implies a response rate of 59.8% of the sexually active people (target population). The prevalence was 4.1% (CI 95%: 3.1-5.8): women: 4% ( CI 95%; 2.8-6.4) and men: 4.3% (CI 95%: 2.9-7.2). The circulating genotype was the E genotype. There was an increase in the risk of *C. trachomatis* infection when barrier contraceptives were not routinely used OR: 4.76 (CI 95%:1.30-17.36) p<0.05.

**Conclusions:**

In our study the prevalence in women resembles those found in other countries in Europe and the prevalence in men is similar to that in women. Screening for *C*. *trachomatis* infection in women would be cost-effective in Spain given the prevalence of *C*. *trachomatis* measured by this study. The use of a condom is the best preventative measure for avoiding STIs in sexually active people.

## Background

*Chlamydia trachomatis* is the most frequently reported of the sexually transmitted infections (STIs) in Europe [1]. *C. trachomatis* genital infection can lead to serious sequelae among women, including pelvic inflammatory disease, tubal factor infertility, ectopic pregnancy and chronic pelvic pain [2] and, as other inflammatory STIs, can also facilitate the transmission of human immunodeficiency virus infection [3].

The US Preventive Services Task Force (USPSTF) [4] recommends screening for *C*. *trachomatis* infection for all sexually active non-pregnant young women aged 24 or younger, and older non-pregnant women who are at increased risk. Although the USPSTF recommendations are being actively followed in some countries, the implementation of screening in Spain has not yet been considered necessary. One of the argued impediments is the lack of accurate numbers regarding the real prevalence of *C*. *trachomatis* infection in the whole of Spain [5]. We have sought to determine this prevalence and in doing so, we were careful to include young males as well, for whom there is scarce data in the European literature. Another planned objective was to delineate the risk factors associated with *C*. *trachomatis* positivity for this target population, information that would be useful for planning future prevention interventions.

## Methods

### Demography

The study was conducted in the borough of Laviana, Asturias, in the north of Spain. It has a population 14,327 distributed in 2 large urban areas and 7 smaller ones. The traditional economic activities are coal mining, agriculture and livestock farming. Education rates are 7.2% university, 51.8% secondary, 26.7% primary and 0.7% illiterate. Immigration accounts for 0.8% of the population. Table 1 shows the census information for young people (15–24 year olds), stratified by age and gender.

**Table 1:** Percentage of sexually active young people in the sexual activity survey, estimated target population and response rate stratified by age and gender

			**Women**								**Men**				
			**Sexual activity survey**				**Prevalence survey**				**Sexual activity survey**				**Prevalence survey**
**Age**	**Census**_**1**_	**RD **_**2**_	**SA **_**3**_	**SA% **_**5 **_**(CI**_**4 **_**95%)**	**Target population**_**6**_	**RD**	**Response rate % **_**7**_	**Age**	**Census**	**RD**	**SA**	**SA% (CI**^**3 **^**95%)**	**Target population**	**RD**	**Response rate %**
15	44	35	4	11.4	5	3	60	15	44	26	4	15.3	7	4	59
16	42	41	21	51.2	22	12	56	16	50	41	17	41.4	21	12	58
17	42	36	23	63.9	27	21	78	17	44	25	13	52	23	8	35
18	55	30	25	83.3	46	25	55	18	62	27	22	81.4	51	22	44
19	54	30	27	90	49	27	56	19	65	36	32	88.8	58	31	54
20	71	48	47	97.9	70	47	68	20	50	27	26	96.2	48	26	54
21	54	33	31	93.9	51	30	59	21	50	25	23	92	46	23	50
22	51	27	26	96.3	49	26	53	22	56	23	23	100	56	23	41
23	72	44	44	100	72	44	61	23	63	33	33	100	63	33	52
24	34	44	44	100	34	42	124	24	45	29	28	96.5	43	28	64
Age group								Age group							
15-19	237	172	100	58.1	138	88	63.9	15-19	265	155	88	56.7	150	77	51.2
				(50.4-65.8)								(48.6-64.8)			
20-24	282	196	192	97.9	276	189	68.4	20-24	264	137	133	97	256	133	51.9
				(94.8-99.4)								(92.6-99.1)			
15-24	519	368	292	79.3	412	277	67.2	15-24	529	292	221	75.7	400	210	52.4
				(75.1-83.6)								( 70.6-80.8)			
Women and Men															
			Sexual activity survey				Prevalence survey								
Age group	Census	RD	SA	SA% (CI 95%)	Target population	RD	Response rate %								
15-24	1048	660	513	77.7	815	487	59.8								
				(74.7-80.9)											

1 Census: population data that referred to a point halfway through the study period (31st May 2011); 2 RD: Responders; 3 SA: Sexually active; 4 CI: *confidence interval;* 5 SA%: Rate of sexually active youth in the Sexual activity survey; 6 Target population: estimated sexually active population in the Census; 7 Response rate %: Rate between sexually active young people who underwent the CT test and the target population.

### Definitions and outcomes measures

**Figure 1:**
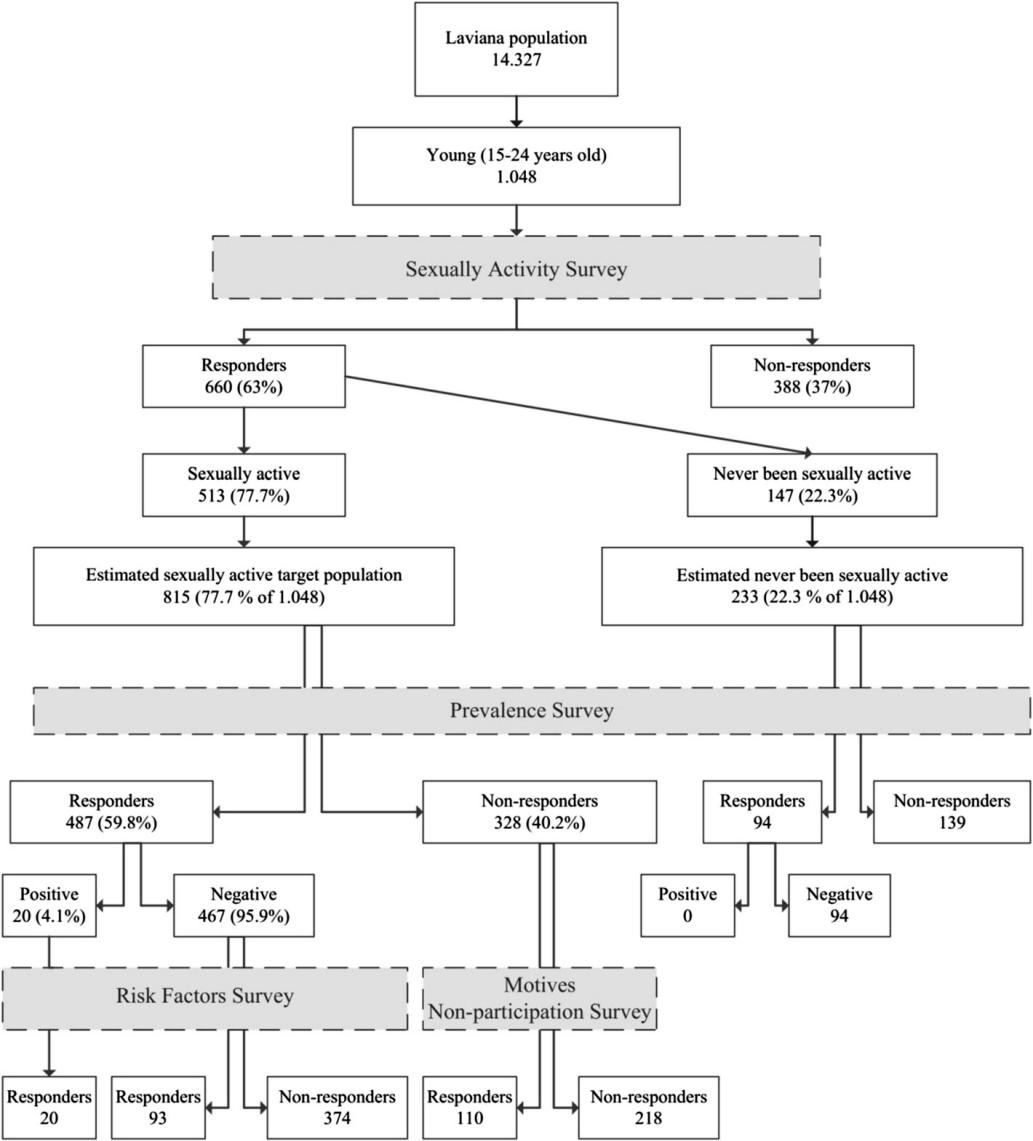
Sequence of study procedures.

Sexual activity was considered as any case of past or ongoing relationship with the practice of sexual (vaginal or anal) penetration or any other behavior that could lead to the transmission of STIs (as fellatio or cunnilingus). The target population was considered as the sexually active people among the 1,048 young (15–24 years old) inhabitants of Laviana and the prevalence was calculated as the proportion of positive tests for *C*. *trachomatis* among the sexually active individuals who underwent the test. The survey was completed in the period between 1^st^ November 2010 and 31^st^ December 2011. Our main outcome measures included the following variables: individuals who underwent the test, positive tests for *C*. *trachomatis*, sexual activity (self-reported as yes or no), age (based on the reported date of birth), gender (female or male), risk factors associated with *C*. *trachomatis* infection (Table 2) and motives for non-participation in the prevalence determination study. Sequence of study procedures are illustrated in Figure 1.

**Table 2:** **Risk factors in *Chlamydia trachomatis *infection**

		**Positive urine test**	**Negative urine test**	**P Value**	**OR (CI 95 %)**
Habitual use of condom					
	No	17	50	0.018	4.76 (1.30-17.36)
	Yes	3	42		
Age in years					
	15-17	2	20	0.25	2.47 (0.53-11.53)
	18-24	8	73		
Last sexual relation with condom					
	No	12	35	0.07	2.44 (0.91-6.57)
	Yes	8	57		
Age group					
	15-19	5	40	0.14	2.26 (0.76-6.75)
	20-24	15	53		
Sexual orientation					
	Heterosexual	20	92	1	NA_1_
	Homo/bisexual	0	1		
New sexual partner in last 3 months					
	0-1	14	69	0.64	1.29 (0.44-3.75)
	>1	6	23		
Early oral contraceptive use					
	No	13	63	0.54	0.70 (0.23-2.17)
	Yes	7	30		
History of abortion					
	No	20	89	0.99	NA
	Yes	0	4		
Travel abroad					
	No	14	63	0.84	0.9 (0.31-2.57)
	Yes	6	30		
Drug use					
	No	20	87	0.99	NA
	Yes	0	6		
Alcohol use					
	No	9	30	0.28	0.58 (0.22-1.55)
	Yes	11	63		
Tobacco use					
	No	16	73	0.88	0.91(0.27-3.03)
	Yes	4	20		
History of urogenital disorders					
	No	15	71	0.83	1.12 (0.37-3.46)
	Yes	5	21		

1 NA: Not Applicable.

Depending on the date when the test was done and the date of birth, a person could have an age that varied during the period of the survey. To neutralize this demographic instability in the calculation of the response rate, we used the population data that referred to a point halfway through the study period (31st May 2011). This could produce a paradoxical effect, such as the capture population being larger that the population of reference, as happened with the group of 24 year old females, where captures ascended to more than 100%.

### Ethics

The study was approved by the Regional Committee for Clinical Research of Principado de Asturias. All the people testing positive for *C. trachomatis* infection and their sex partners were sent to ambulatory care clinics for treatment and control.

### Sexual activity and target population survey

To determine the size of the target population (sexually active people) of our prevalence study, a questionnaire on age, gender and sexual activity was carried out on the young people when they underwent the test, and for the remaining people who had not undergone the test, the sexual activity survey was carried out by post. So finally all young people between 15 and 24 years old living in Laviana were invited to participate in the study.

### Capture strategy

In order to increase the level of participation, this study was approved by the Health Council and was publicized within the whole population and in the media (press, radio). As STI-related testing can create noticeable barriers, due to fear of stigmatization or reluctance to share information about sexual activity and preferences, we employed the method of Shafer [6] to increase screening rates. In detail, we designed the following 7 capture strategies. 1) We conducted a capture strategy in the whole population instead of only in a sample group. 2) Urine samples were collected from sexually active people and people who have never been sexually active. 3) The *C*. *trachomatis* test was conducted on all urine samples in order to eliminate ethical considerations and increase the number of captured positives that would have been lost on account of false declaration by the participants. 4) Informative workshops were organized in all the secondary schools in Laviana where the home self-sampling kit was distributed. 5) Opportunistic capture was also employed in the local health centre among young people who attended for other causes. In this setting, the sexual activity survey was conducted by the resident physicians and nurses. 6) When all other methods failed, letters were sent by post to all young 15–24 year olds that had not been tested. In the case of no reply, the individuals were contacted by phone as a last resort. These activities were carried out by the clerical staff in the Primary Care Center, who were stimulated with incentives (regular feedback at face-to-face meetings with the investigating team about their screening rates, days off and financial incentives) and assertive reinforcement: “*Chlamydia Screening Champions*” (the success of this study depended on their active cooperation). 7) The clerical staff handed out the home self-sampling kit, and to ensure confidentiality young people could collect the home self-sampling kit and return it to various places in the town, namely pharmacies and the Youth Information Office, so they could avoid coming to the Health Center and being recognized by other patients.

### Testing kit, RT-PCR and genotype characterization

The home self-sampling kit included: the VERSANT® system for urine transport, information about how to collect the urine sample, a questionnaire about their sexual activity, personal data and an explicit consent form complete with envelope. The urine samples were collected and transported with the VERSANT® system. Then they were sent to the Microbiology Laboratory Service at the “Hospital Universitario Central de Asturias”, where they were labeled and identified by barcode, and refrigerated for 14 to 21 days at +4°C according to the manufacturer’s guidelines. The automatized real time PCR (kPCR, Versant CT/GC DNA 1.0, Siemens Healthcare Diagnostics) uses single tube detection for *C*. *trachomatis* and *Neisseria gonorrhoeae* and internal control. For genotype characterization, a fragment of the *omp*A gene was amplified according to the report by Lysen [7]. In detail, amplicons were purified by using a Montage DNA Gel Extraction Kit (Millipore, Bedford, MA, USA) and sequenced with the BigDye Terminator Cycle Sequencing Kit (Applied Biosystems, Foster City, CA, USA). The individual sequences were aligned using the Clustall-W2 program (European Bioinformatics Institute).

### Risk factors survey

Those who answered positively to active sexual activity and underwent the urine test filled out a form about risk factors for *C. trachomatis* positivity. The survey was carried out in a Health Centre on the subjects who gave a positive result for *C. trachomatis* in the test, and by an anonymous questionnaire on the subjects who gave a negative result.

### Assessment of the prevalence survey’s quality

In order to account for assignment bias an additional survey was carried out at the end of the prevalence study for non-responders. This survey looked into the motives for non-participation in the prevalence study, it was anonymous and carried out in secondary schools during sex education workshops in the case of young people aged 15–17 years old and by post for subjects between 18 and 24 years old.

### Statistical analysis

We calculated the prevalence in sexually active people using the Wilson score to estimate the confidence interval (95%). We chose the Wilson score due to its design for lower prevalence studies. For the risk factor survey, the STATCAL program (EPIINFO v7, CDC, Atlanta, USA) was used for bivariate over multivariate analysis. For binary and multinomial logistic regression, and analysis of the sensitivity of assumed models we used SPSSv15 software (SPSS Inc, Chicago, IL, USA) together with an analysis of the sensitivity of different models. Estimation of punctual association magnitude was performed with the Odds ratio from the exp (B) of the model to interval estimation with the OR confidence interval.

## Results

### Sexual activity and target population survey

**Figure 2:**
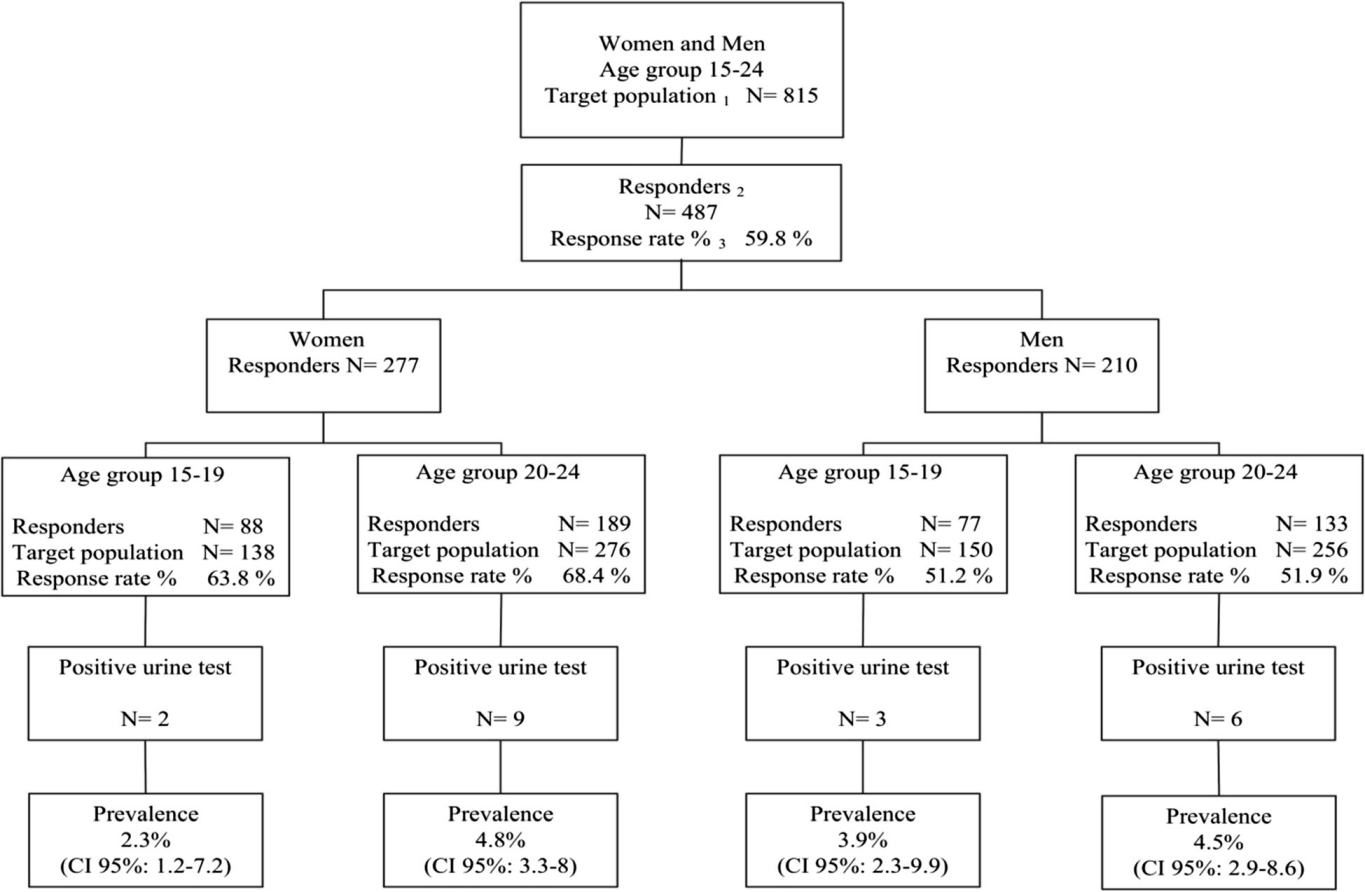
Prevalence survey results: distribution per age and gender.

Six hundred and sixty young people (63%) among 1048 young inhabitants of Laviana answered questions about their sexual activity and gender. Out of these 660 young people, 513 were sexually active (77.7%), which implies an estimated sexually active target population of 815 young 15–24 year olds (Figure 2).

In Table 1, the percentage of sexually active young people in the sexual activity survey, the estimated target population, the people who underwent the test and the response rate of the prevalence survey are shown stratified by age and gender.

The prevalence survey included 487 people with sexual activity, which implies a response rate of 59.8% of the target population. The distribution by age group and gender of the response rate was: a) Women aged 15–24 years 67.2% (15–19 years 63.8% and 20–24 years 68.4%); b) Men aged 15–24 years 52.4% (15–19 years 51.2% and 20–24 years 51.9%).

### Prevalence survey

The survey to determine the prevalence of *C*. *trachomatis* infection in the community included 581 people aged 15–24 years, of whom 487 were sexually active and 94 have never been sexually active. Among the 487 sexually active people, twenty urine samples were found to be positive. All samples from the population without sexual activity were negative. In Figure 2, the whole testing algorithm for the prevalence survey is depicted graphically. The overall prevalence was 4.1% (CI 95%: 3.1-5.8): women 4% (CI 95%: 2.8-6.4) and men 4.3% (CI 95%: 2.9-7.2). The distribution by age groups and gender was: a) Women, aged 15–19 years 2.3% (CI 95%: 1.2-7.2) and 20–24 years 4.8% (CI 95%: 3.3-8.0). b) Men aged 15–19 years 3.9% (CI 95%: 2.3-9.9) and 20–24 years 4.5% (CI 95%: 2.9-8.6).

The phylogenetic analysis of *C*. *trachomatis* from 17 positive samples (85% of the positive urine samples) showed that the genotype circulating in this population was E (100%).

### Motives for non-participation in the prevalence determination survey

One hundred and ten of the 328 sexually active people who had not undergone the test declared their reasons for not participating in the prevalence study. 38.1% were unaware of the existence of the investigation, 28.6% did not see their participation as necessary as they did not have “risky” sexual relations, 7.2% said they were not interested in taking the test, 6.8% said they forgot they had an appointment with the doctor, 6.7% said they were on holiday when it was their turn to do the test, 4.8% did not agree with the nature of the study, 3.1% did not participate due to concern that they might test positive and have to communicate this to their partner, 3.1% considered STIs to be “a myth” and 1.6% did not trust the confidentiality of the test.

### Risk factors for *C*. *trachomatis* positivity

Ninety three sexually active 15 to 24-year-olds with negative urine test and 20 with positive urine test completed the questionnaire on the predisposing risk factors for *C*. *trachomatis* positivity (Table 2).

The risk factor of routine sexual activity without the use of condoms showed a statistically significant association with *C*. *trachomatis* infection: OR: 4.76 (CI 95%:1.30-17.36) p<0.05. There was no statistically significant association with other variables.

## Discussion

Because of the large burden of disease and risks associated with infection, the USPSTF recommends screening for *C*. *trachomatis* infection for all sexually active non-pregnant young women aged 24 or younger and older non-pregnant women who are at increased risk [4]. The threshold population prevalence of *C*. *trachomatis* infection over which the evaluations were cost-effective varied from 3.1 to 10%. Screening can be cost-effective at a prevalence as low as 1.1%, when age is used to select women and nucleic acid amplification based tests are carried out on urine samples, although the large numbers of unnecessary tests diminishes effectiveness [8].

There are scarce data regarding the prevalence of *C. trachomatis* infection in Spain, all of them from risk population attending in STI clinics. Corbeto *et al.* studied people under 35 years-old attending in sexual health clinic and concluded that overall *C. trachomatis* prevalence was 4% , but it was higher in those under 25 years-old (5.8%) and higher than in our non-selected population (4.1%) [9]. Nogales *et al*. studied young and adults attending in a clinic for STI, 50% of them belonged to groups engaged in high risk sexual practices as commercial sex workers, MSM, users of prostitution, and the prevalence was 6% (4.3% in women and 7.8% in men) [10]. Similar prevalence (5.9%) was found by Folch *et al*. in immigrant female sex workers, but these data cannot be compared with our study due to differences in population characteristics [11].

Comparisons with prevalence data previously reported by other countries are not simple, owing to the use of different age groups to calculate rates. In our survey, the *C. trachomatis* prevalence in women (4%) resembles those found in other countries in Europe. In a sexual survey in France in 2006 [12], the prevalence of *C*. *trachomatis* in the national population in women in the 18–24 year group was 3.6% (CI 95%: 1.9-6.8) and in men 2.4% (CI 95%: 1–5.7). In the UK, in the second National Survey of Sexual Attitudes and Lifestyles [13] the prevalence of *C*. *trachomatis* in women in the 18–24 years group was 3% (CI 95%: 1.7-5) and in men 2.7% (CI 95%: 1.2-5.8). In Slovenia, considering the prevalence in the captured general population (1999–2011), the prevalence in women and men in the 18–24 years group was found to be 4.7% (CI 95%: 2.5-8.5), and was the same in both genders [14]. Confidence intervals for these prevalence estimates were wide, with overlapping 95% confidence intervals, and the differences between countries were not statistically significant.

In the USA, where substantial racial/ethnic disparities in *C. trachomatis* infection exist, the prevalence in women is higher for 15–19 year olds and lower for 20–24 year olds when compared to the results in our survey. According to data from the National Health and Nutrition Examination Survey 1999–2008 (NHANES) [15], the *C*. *trachomatis* prevalence among sexually active women in the 14–19 age range was 6.8% (4.4% among non-Hispanic Caucasians and 16.2% among non-Hispanic Afro-Americans) and in the 20–24 age range it was 3.2% (1.3% among non-Hispanic Caucasians and 12.1% among non-Hispanic Afro-Americans). In our study the prevalence of *C*. *trachomatis* in the 20–24 years group (women 4.8% and men 4.5%) is higher than that seen in the 15–19 years group (women 2.3% and men 3.9%). In the 15–19 years group the level of sexual activity was especially low (women 58.1% and men 56.7%) and in the 20–24 years group it was women 97.9% and men 97% (Table 1). The prevalence of *C*. *trachomatis* infections among young adults (aged 18–26 years) in the USA who participated in the nationally representative National Longitudinal Study of Adolescent Health during 2001–2002, was: global prevalence 4.19%; women (4.74%) and men (3.67%) [16].

Our prevalence in men (4.3%) was similar to that in women (4%). Increasing efforts in partner notification and their treatment may contribute at least as much to the control of *C*. *trachomatis* infection as increasing screening coverage rates [17].

Our results, in both men and women, suggest that prevention of *C*. *trachomatis* with routine condom use could lead to a reduction in the observed *C*. *trachomatis* infection rates. However, as non-compliance with safe sex practices is commonly seen in young age groups and complications of a *C*. *trachomatis* infection are sometimes severe, with infertility and major sequels, universal free screening for young sexually active people in the health sector would aid the immediate recognition of the infection and thus facilitate action towards implementing the available effective therapy.

A response rate of 60% has been used as the threshold of acceptability by some and is valid as a measure of survey quality; however 60% is only a “rule of thumb” that masks a more complex issue. Non-response bias is more useful for understanding survey limitations [18]. This non-response can potentially bias the prevalence estimates under 2 conditions: if the response rate varies according to an observed attribute, such as age, race, gender or sex, which is associated with prevalence or if the non-respondents have a different pattern of prevalence from respondents with similar observed attributes [13]. But once the variable of age was adjusted according to sexual activity by means of a stratified analysis, it showed us that there was no significant difference with regards to participation related to age (Table 1). All this, being considered with the analysis of the motives for non-participation, with a response rate of 59.8% in our prevalence determination study, showed us that the distribution of the captured population was not distorted and comparable to the total reference population.

There are some limitations in our survey. Only 93 of the 467 sexually active young people with a negative urine test answered the survey on risk factors for *C*. *trachomatis* infection. Due to the fact that the young people had to give their names in the prevalence survey, we were worried that the numbers participating in that prevalence survey could have been reduced, as the young people would not want to be identified on account of their reluctance to report their sexual activity and preferences. Therefore, we made the second test, the sexual risk factor survey, anonymous and at a later date. This second survey had low numbers of participants showing the young people’s reluctance to report their sexual activity and preferences. Another limitation is the target population (the sexually active population), an unknown quantity, was determined by an initial survey, and therefore it must be considered as an estimation. Other limitations are the potentially inaccurate reporting of sexual behaviour data (social desirability bias), response rate and lack of information about non-responders, and lack of generalisability of these results.

The generalization of these prevalence values to other places in Spain could be possible but considerations about sample size, geographical limitations, lack of socioeconomic stratification and other factors, should be considered. We think that this is the first study in Spain to determine the communitary prevalence of *C. trachomatis* genital infection in young people.

## Conclusions

The prevalence in our survey resembles those found in other countries in Europe. Screening for *C*. *trachomatis* infection in women would be cost-effective in Spain given the prevalence of *C*. *trachomatis* measured by this study. The use of a condom is the best prevention measure for avoiding STI in sexually active people.

## Abbreviations

STI: Sexually transmitted infection; USPSTF: United States Preventive Services Task Force.

## Competing interests

The authors declare that they have no competing interests.

## Authors’ contributions

C-FB initiated the collaborative project, designed data collection tools, implemented the trial, monitored data collection for the whole trial, analyzed the data, and drafted and revised the paper. P-ML and P-SL analyzed the data and laboratory studies, and revised the paper. L-OG analyzed the data and statistical analysis plan and, drafted and revised the paper. MJ-MM wrote the statistical analysis plan, cleaned and analyzed the data, and drafted and revised the paper. F-V initiated the collaborative project, designed data collection tools, implemented the trial, monitored data collection for the whole trial, analyzed the data, and drafted and revised the paper. The Chlamydial Primary Care Group participated in the field study. All authors read and approve the final manuscript.

## Pre-publication history

The pre-publication history for this paper can be accessed here:

http://www.biomedcentral.com/1471-2334/13/388/prepub

## Chlamydial Primary Care Group

Magdalena Álvarez- Sánchez, Juan Jesús Hidalgo- Rodríguez, Camila Martínez -Rivera, Maria Lema-González, Pablo Fernández -Menéndez, Marta Fernández- Moran, Francisco Carreño-Alonso, María Luisa Junquera- Llaneza, Santiago Fernández-Menéndez, María del Mar García-Cuesta, José Antonio Varela- Uría, Paula Nieto- Barros and María del Carmen López- Sánchez.
